# Regulation of Ribosomal Proteins on Viral Infection

**DOI:** 10.3390/cells8050508

**Published:** 2019-05-27

**Authors:** Shuo Li

**Affiliations:** Institute of Plant Protection, Jiangsu Academy of Agricultural Sciences, Nanjing 210014, China; lishuo@jaas.ac.cn; Tel.: +86-025-84390394

**Keywords:** ribosomal proteins, viral infection, regulation, antiviral therapeutics

## Abstract

Ribosomal proteins (RPs), in conjunction with rRNA, are major components of ribosomes involved in the cellular process of protein biosynthesis, known as “translation”. The viruses, as the small infectious pathogens with limited genomes, must recruit a variety of host factors to survive and propagate, including RPs. At present, more and more information is available on the functional relationship between RPs and virus infection. This review focuses on advancements in my own understanding of critical roles of RPs in the life cycle of viruses. Various RPs interact with viral mRNA and proteins to participate in viral protein biosynthesis and regulate the replication and infection of virus in host cells. Most interactions are essential for viral translation and replication, which promote viral infection and accumulation, whereas the minority represents the defense signaling of host cells by activating immune pathway against virus. RPs provide a new platform for antiviral therapy development, however, at present, antiviral therapeutics with RPs involving in virus infection as targets is limited, and exploring antiviral strategy based on RPs will be the guides for further study.

## 1. Introduction

A ribosome is a type of intracellular machinery responsible for protein biosynthesis in all cells; this process from RNA to protein in the genetic central dogma is also known as “translation”. Outside of its catalytic role, the ribosome is also a translational regulator during protein synthesis. Ribosome components include ribosomal proteins (RPs) and ribosomal RNA (rRNA). In eukaryotes, the biogenesis of ribosomes occurs within the nucleolus and requires the coordinated assembly of 4 rRNAs (5S, 5.8S, 18S, and 28S rRNAs) and 80 (79 in yeast) RPs [1,2]. The eukaryotic ribosome, termed 80S ribosome, is composed of two subunits. The 40S small subunit has the decoding function, which is composed of 18S rRNA and 33 RPs [2]. The 60S large subunit catalyzes the formation of peptide bonds, which is composed of 5S, 5.8S, 28S rRNAs and 47 (46 in yeast) RPs [2]. Apart from being components of ribosomes, RPs also play essential roles in ribosome biogenesis, in which RPs as RNA chaperones stabilize rRNAs and promote their correct folding for assembly of ribosomal subunits [3,4]. Increasing evidence has demonstrated the extra-ribosomal functions of RPs. RPs could regulate many fundamental life processes involving the cell cycle, cell proliferation, apoptosis, tumorigenesis, genomic integrity and development, etc. [4,5,6,7].

The viruses are the small infectious pathogens that replicate only inside the living cells. Viruses can infect all types of living organisms, from animals and plants to microorganisms. As the smallest infectious pathogen, viruses have limited genomes, so during viral infection, viruses must employ a variety of host cellular factors in order to survive and produce new viral particles. Specifically, it is now appreciated that RPs play critical roles in the life cycle of viruses. In virus-infected cells, virus may usurp an endogenous translation pathway [8]. After infection by some viruses (for example herpes viruses), translation of cellular mRNA is often selectively suppressed, whereas translation initiation of RPs mRNA increases and persists late [9,10]. It is a result of the virus–host interaction, for preferentially producing RPs to maintain viral propagation. In these related functions of RPs, some are ribosome-dependent, mainly participating in viral protein biosynthesis, and the other are independent of the ribosome, involving in the regulation of virus infection in host cells. In this review, the most relevant studies are summarized, and the author will focus on the roles of RPs in regulation of viral replication and infection in host cells and discuss RPs as potential targets for antiviral therapeutics.

## 2. Positive Regulation of Ribosomal Proteins on Viral Infection

### 2.1. Viral Internal Ribosome Entry Site (IRES) Hijacks the Ribosome

**Figure 1:**
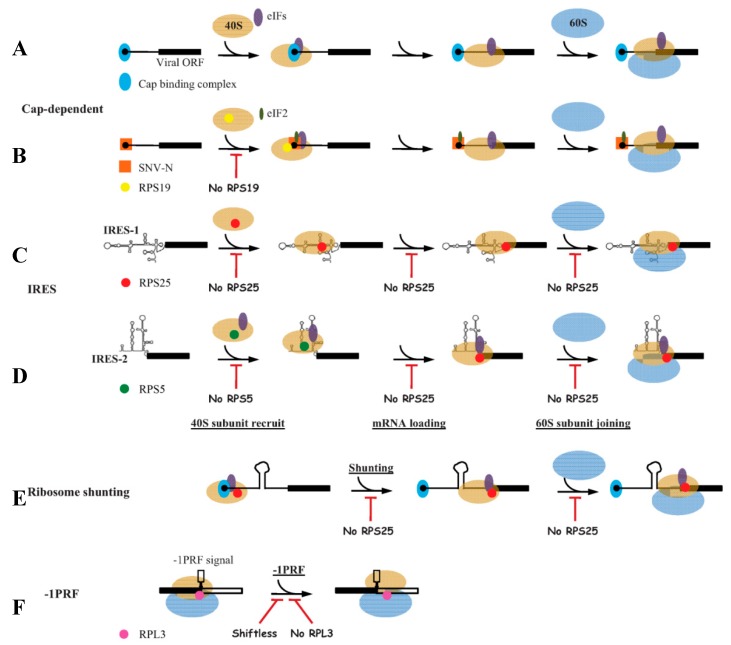
Models for the roles of ribosomal proteins (RPs) in regulating viral mRNA translation initiation. (**A**) Cap-dependent translation initiation, in which cap-binding complex (eIF4F) and eukaryotic initiation factors (eIFs) are required; (**B**) N protein of *Sin nombre hantavirus* (SNV) interacts with RPS19 to recruit 40S subunit to 5′ cap, directly mediating viral mRNA translation initiation independently of cap-binding complex; (**C**) The internal ribosome entry site 1 (IRES-1) (from IGR of *Dicistroviridae* family) depends on RPS25 for 40S subunit recruitment, mRNA loading and 60S joining, and no eIFs are required in this process; (**D**) IRES-2 (from hepatitis C virus) relys on RPS5 to bind 40S subunit with the participation of eIFs, and RPS25 is required in subsequent mRNA loading and 60S joining process; (**E**) In ribosomal shunting, RPS25 is also indispensable; (**F**) In programmed -1 ribosomal frameshifting (-1PRF), RPL3 controls translational fidelity. Shiftless inhibits -1PRF via interacting with RPS27A, RPL11 and release factors. Legends are annotated in the figure. eIFs (purple) refer to various eIFs, which are different in different steps. Underlined text indicates different steps in the viral translation. Italics “No RPS25” indicates the depletion or knockout of this RP, and the rest is the same. Red T-shaped symbol represents the block of the step. The secondary structures of IRES-1 and IRES-2 are schematic, and do not represent the real stem-loops.

The predominant translation initiation pathway of cellular mRNA is cap-dependent, which requires recognition of mRNA 5′ cap by the eukaryotic initiation factor 4E (eIF4E). eIF4E interacts with eIF4G and eIF4A to form the cap binding complex (eIF4F), recruiting the 40S subunit to the 5′ end of mRNA (Figure 1A) [11]. However, many viral mRNAs have no 5′ cap structures. The cap-snatching from host mRNA is an effective strategy [12,13], while some RNA viruses utilize a cap-independent manner to recruit the ribosome, which is mediated by the internal ribosome entry site (IRES) [14,15,16].

IRES is a *cis*-element in 5′ untranslated region (UTR) of viral mRNA, and it can recruit ribosomes to initiate the synthesis of viral proteins [17,18]. According to the secondary structure, required initiation factors (eIFs) and location of the start codon relative to IRES, viral IRESs are functionally divided into four groups (IRES 1–4) [19]. IRES-1, from the intergenic region (IGR) of *Dicistroviridae* family that infects invertebrate, is the most streamlined IRES, because it recruits the 40S and 60S subunits to form functional 80S complexes in the absence of any eIFs [19,20,21]. Cricket paralysis virus (CrPV), Plautia stali intestine virus (PSIV) and Taura syndrome virus (TSV) are typical members in this group [19]. IRES-2 needs a subset of the canonical eIFs (eIF3, eIF2) and Met-tRNA to bind to 40S subunit [19]. This group is mainly from *Flaviviridae* family that infects mammal, with Hepatitis C virus (HCV) and Classical swine fever virus (CSFV) as typical members [19,22,23]. For IRES-3 (including Encephalomyocarditis virus and Foot-and-mouth disease virus, etc.) and IRES-4 (including poliovirus and rhinovirus, etc.), besides some canonical eIFs and Met-tRNA, additional proteins named IRES trans-activating factors (ITAFs) are also required to bind ribosome [14,15,19,24]. However, even in the simplest system, an intricate reaction network regulates translation initiation, in which RPs are essential [18,25].

Although IRES-1 and IRES-2 both induce a conformational change in the ribosome upon binding [23,26,27], their binding sites to 40S subunit are different. IRES-1 binds to the intersubunit surface of 40S subunit occupying the peptidyl (P) and exit (E) sites [27], and IRES-2 binds to the solvent side of 40S subunit and occupies E site [28]. The binding to 40S subunit of IRES-2 is regulated by RPS5 with its β-hairpin structure, independently of RPS25 (a RPS5 neighbor situated in the head domain of 40S subunit) (Figure 1D) [29,30,31,32,33]. Whereas IRES-1 depends on RPS25 for binding to 40S subunit (Figure 1C) [18,31,34], in which RPS5 could maintain the accuracy of translation in eukaryotes [35]. RPS25 is non-essential in viral cap-dependent translation, but it plays a critical role in all viral IRES-mediated translation. The propagation of HCV and poliovirus is impaired in cells depleted of RPS25 [31]. This is because, after 40S subunit recruitment, all groups of IRESs rely on RPS25 for efficient translation (Figure 1C,D) [31,34,36]. RPS25 likely functions in a downstream step, such as loading of viral mRNA into 40S subunit, start codon recognition, or 60S subunit joining [31,36]. When cap-dependent translation initiation is hindered, the 5′ leader of human immunodeficiency virus type 1 (HIV-1) genomic RNA can fold into IRES to recruit 40S subunit and drive translation initiation of viral Gag protein, in which the activity of IRES is also dependent on RPS25 [37]. RACK1, as ribosome scaffold protein, is also required for viral IRES-2-mediated translation, and the effect of RACK1 may associate with eIF3 to assemble a translation preinitiation complex [38]. In addition, it is demonstrated that the intracellular level of 40S subunit plays a key role in facilitating HCV translation over host translation, and the translation initiation of HCV IRES and host mRNA possess different susceptibility for reduction of 40S subunit [39]. RPS2 (p40), RPS3a, RPS14 and RPS16 can interact with HCV IRES [40,41], which might be one of the reasons that HCV IRES is more sensitive to 40S subunit level, and the roles of these RPs in translation initiation of viral RNA require further explanation.

### 2.2. Ribosome Shunting and Programmed -1 Ribosomal Frameshifting

Ribosome shunting is one of alternate mechanisms of virus initiating translation. In the process, 40S subunit is recruited to the 5′ end of viral RNA through a cap-dependent manner. After scanning 5′ UTR or translating a short open reading frame (ORF), 40S subunit jumps from a shunt donor region to the downstream acceptor region and recognizes a renewed AUG to initiate protein synthesis, bypassing the intermediate secondary structure regions of transcript without scanning through it [42]. Members exploiting ribosome shunting strategy include Cauliflower mosaic virus (CaMV), Rice tungro bacilliform virus, Sendai virus and adenovirus, etc. [42,43,44,45]. At present, the shunting mechanism is still not well understood. It has been reported that 40S subunit is fastened to mRNA by either base pairing with 18S rRNA or binding to eIF3 [46,47]. Furthermore, ribosome shunting by adenovirus tripartite leader is impaired in RPS25-deficient cells, which suggests that RPS25 is also required for ribosome shunting (Figure 1E) [31]. RPS25 may perform a common function in IRES and ribosome shunt mediated translation initiations [31].

Programmed -1 ribosomal frameshifting (-1PRF) is also a widely used translation recoding mechanism by virus, wherein a part of translating ribosomes slip back one nucleotide site so that the translation continues in a new ORF [48]. Through this mechanism, virus can translate one mRNA template to produce two different proteins, which expands the utilization of viral limited genetic information. The typical case of the mechanism is that HIV-1 expresses Gag-Pol polyprotein from the Gag-coding mRNA through -1PRF. When HIV-1 Gag is translated to the end of its ORF, a fraction of ribosomes shifts the reading frame to -1 site via the recognition of -1PRF signal, resulting in the production of Gag-Pol [49,50]. The ratio of Gag to Gag-Pol is strictly maintained for efficient virus assembly and maturation [51,52]. Recently, the -1PRF regulatory mechanism of HIV-1 is well elucidated. The host factor Shiftless is identified as an inhibitor of -1PRF, which interacts with -1PRF signal of HIV-1 mRNA and translating ribosomes and causes premature translation termination at the frameshifting site (Figure 1F) [53]. In this process, translation release factor eRF1 and eRF3 are required, as well as eS31 (RPS27A) and uL5 (RPL11), which interact with Shiftless. Upon the conformational rearrangements and intersubunit rotations, the two RPs respectively from 40S and 60S subunits join forces to sustain the association of Shiftless with the ribosome and viral RNA [53]. In yeast, RPL3, locating at the peptidyl transferase center, is responsible for translational fidelity of -1PRF (Figure 1F), and the *RPL3* mutations lead to rapid loss of M1 killer virus [54].

### 2.3. Phosphorylation of Ribosomal Proteins

Accumulating studies indicate that RPs are subject to phosphorylation in response to virus infection [55,56,57]. The phosphorylation of RPS6 is a famous representative and has attracted much attention since its discovery stimulated by Vaccinia virus in 1976 [58]. A flood of viruses has been shown to induce phosphorylation of RPS6, such as Pseudorabies virus (PRV), Herpes simplex virus-1 (HSV-1), Polyoma virus, Simian virus 40, Avian sarcoma virus, tumor viruses, etc. [59,60,61,62]. RPS6 kinase (RPS6K) from host cells is responsible for this modification [57,63,64], while some viruses can produce a protein with kinase activity. B1R protein kinase of Vaccinia virus can phosphorylate RPS2 [65,66], and tyrosine-specific protein kinase of Abelson murine leukemia virus enhances RPS6 phosphorylation by activating RPS6K and/or inactivating RPS6 phosphatase [67]. However, despite a large number of reports on the increasing phosphorylation of RPs induced by viral infection, the role of this modification on virus infection is still poorly understood.

Notably, this modification occurs during the early stage of virus infection and in both the cytosol and the nucleus [55,57,59]. One viewpoint is that RPS6 phosphorylation is a normal physiological response of cell to numerous internal and external stimuli. It has been demonstrated that RPS6 is subject to phosphorylation in response to a series of physiological (such as stresses, nutrients, lipid, hormones and mitogenic stimulation), pathological, and pharmacological stimuli [57,68]. Viral infection is only one of numerous stimuli, and RPS6 phosphorylation is a nonspecific response to virus. The phosphorylation of RPS6 still occurs in cells transformed by completely inactivated PRV, suggesting that expression of viral genome is not required for virus eliciting phosphorylation [69]. When culture medium from cells infected by PRV is freed of virus and added to confluent fibroblasts, RPS6K is rapidly activated, which indicates that RPS6 phosphorylation in virus-infected cells is a consequence of the production of cell factors [64]. These factors may initiate the metabolic programme for cellular growth [64]. RPS6 phosphorylation is always correlated with efficient translation of RPs mRNA [9], so it might be a cellular response to numerous stimuli through regulating translation of various RPs. The other view is that RPS6 phosphorylation is required for replication and accumulation of many viruses. NsP2 protein of alphavirus, an essential component of the viral replication complex, interacts with RPS6. A reduction of RPS6 level diminishes the expression of viral mRNA, but does not dramatically impair host cellular translation, indicating that alphavirus alters ribosome via RPS6 phosphorylation and the alteration may contribute to differential translation of host and viral mRNA [70]. In Kaposi’s sarcoma-associated herpesvirus (KSHV)-infected cells, the latency-associated nuclear antigen (LANA) is a viral maintenance factor, and RPS6 can interact with LANA to maintain its extraordinary stability [71]. Interestingly, for plant viruses, the requirement for RPS6 phosphorylation differs for diverse viruses with different translation initiation strategies [72,73]. The cap-independent Turnip mosaic virus (TuMV) requires RPS6 and RPS6K for viral accumulation in plant cells, in which the viral genome-linked protein (VPg) of TuMV interacts RPS6K in both the cytoplasm and the nucleus [72,73]. Conversely, accumulation of Tobacco mosaic virus (TMV) with the 5′ m^7^Gppp cap and the Ω leader is independent of RPS6 and RPS6K [72,73]. The molecular mechanisms underlying the diverse effects of RPS6 phosphorylation on virus infection in cells needs further explanation.

### 2.4. Interaction Between Viral Proteins and Ribosomal Proteins

Viral proteins, regardless of structural and nonstructural proteins, interacting with diverse RPs are widely reported. Just an N-terminal protease (N^pro^) from pestivirus, there are 26 RPs interacting with it [74], and P1 of Tobacco etch virus (TEV) interacts with 17 RPs in *Nicotiana benthamiana* [75]. Of course, the significance of interaction still needs to be identified. The characterized roles (in Table 1) of the interactions on positive regulation of virus infection can be roughly divided into 3 classes: 1) facilitating viral translation, 2) involving in virus assembly and replication, and 3) as viral receptor. Class 1 belongs to ribosomal function, in which *Sin nombre hantavirus* (SNV) N-RPS19 interaction is a typical case. Through interacting with RPS19, SNV-N could combine with the 40S subunit, mRNA cap and unphosphorylated eIF2 to form the 43S pre-initiation complex, replacing the eIF4F complex to directly mediate viral mRNA translation initiation (Figure 1B) [76,77,78]. RPL18 is also a well-known critical factor due to its interacting with many viral proteins. RPL18 of *Arabidopsis thaliana* interacts with P6 of CaMV in a complex consisting of several RPs and eIF3, which is required for translational transactivation of CaMV [79,80]. RPL18 is found to be incorporated into Ebola virions, and reduced expression of *RPL18* effectively inhibits Ebola virus infection in 293T cells [81]. It is demonstrated that N of Rice stripe tenuivirus interacts with RPL18 of insect vector and silencing of *RPL18* significantly reduces viral translation and replication in insect vector [82]. Interaction between NS1 of Dengue virus (DENV) and RPL18 in human hepatic cells (Huh-7) exhibits a similar significance [83]. The above researches describe the upregulating role of RPL18 on virus translation. Class 2 and 3 is beyond ribosomal function. For example, RPL7, as a HIV-1 Gag helper factor, exhibits a potent DNA/RNA chaperone activity and contributes to Gag-mediated virus particle assembly [84]. In many cases, RPs exercise these functions depending on the specific localization outside of ribosome. After entering into the nucleus, RPL22 translocates from the nucleoli to the nucleoplasm to interact with HSV-1 ICP4 for regulating viral DNA synthesis and late gene expression [85]. RPS2 as membrane receptor binds envelope protein E of DENV and Yellow fever virus (YFV) [86]. The helper component proteinase (HCpro) of potyviruses is a multifunctional protein [87]. As a suppressor of antiviral RNA silencing, HCpro interacts with ARGONAUTE1 (AGO1) and RPL18 to form complexes to relieve viral RNA translational repression from RNA-induced silencing complex (RISC) [88]. HCpro can interact with translation factors both eIF4E and eIF(iso)4E [89], while RPL10 (P0) promotes translation of Potato virus A by interacting with VPg and eIF(iso)4E [90]. It is reported that HCpro of TEV inhibits translation, but this inhibition is relieved by TEV P1 interacting with plant ribosome [75]. Besides, HCpro is also a vector transmission helper factor, mediating the retention of virions to aphid mouthparts. In the process, RPS2, as the potential laminin receptor precursor, binds HCpro of TEV to assist virus transmission [91].

### 2.5. Ribosomal Proteins that Promote Viral Infection Without Directly Interacting with Viral Proteins

Using gene silencing or knockout techniques, many RPs that enhance viral proliferation are identified, but the direct interaction between these RPs and viruses is not reported. Knockdown of RPLP1 and RPLP2 strongly reduced early DENV protein accumulation, while global protein synthesis is relatively stable in cells, suggesting a requirement for RPLP1/2 heterodimer in viral translation [100]. RPL40 is required for cap-dependent translation initiation of Vesicular stomatitis virus, Measles virus and Rabies virus by acting in the 80S formation step [8]. A Hepatitis E virus recombinant strain with RPS17 insertion in the hypervariable region of viral ORF1 endows enhanced virus replication activity, indicating the importance of RPS17 for virus infection [101]. The interaction of eIF-5A with HIV-1 Rev trans-activator mediates the nuclear export of viral unspliced mRNA [102]. In the process, eIF-5A utilizes the 5S rRNA cellular transport system through partnering with RPL5, a central component of 5S rRNA export system [102]. Despite the differential requirement of RPS6, the accumulation of TMV, TuMV and Tomato bushy stunt virus (TBSV) is dependent on RPL19, RPL13, RPL7 and RPS2, regardless of their translation strategies [73].

After some virus infections, translation of host other mRNA is often suppressed, but RPs mRNA translation and ribosome biogenesis increase [9,10]. This phenomenon is considered as a result of the virus–host interaction. The modulation of ribosome biogenesis in the nucleolus by coronaviruses might be a better case to uncover the mechanism. The nucleolus is a dynamic sub-nuclear structure, which is involved in ribosome subunit biogenesis, modulation of cellular growth and the cell cycle, etc. [103,104]. N protein of Avian infectious bronchitis coronavirus (AIBV) localizes to the nucleolus in a cell cycle dependent manner and interacts with fibrillarin and nucleolin, which are two major components of the nucleolus involved in ribosome biogenesis [105,106]. The nucleolus entry of AIBV N protein results in the specific changes of the nucleolar proteome and delay of the cell cycle, which promotes host ribosome biogenesis and facilitates indirectly translation of viral mRNAs [106,107,108].

### 2.6. Ribosomal Proteins in Replication and Transcription of Viral Genome

The aforementioned RPs functions mainly focus on the domination and regulation of viral translation and assembly. Though not so large in number, some RPs have been shown to take part in viral genome replication or transcription in host. RPS27 is a metallopanstimulin containing a C4-typezinc finger peptide (ZFP) motif that can bind zinc and other transition metal ions, such as iron or copper [109,110]. RPS27 is not required for the function of ribosome, but it plays a critical role in the life cycle of various viruses through regulating viral nucleic acid replication and gene transcription [109,111]. When RPS27 is disrupted in host cells, which is disposable for the host, the replication and infectivity of numerous viruses is abolished, including Influenza A virus (IVA), Drosophila C virus, DENV, HCV, Sindbis virus, Border disease virus, etc. [109,111,112,113]. It is reported that RPS1 is a subunit of Qβ replicase involving in RNA replication of Coliphage Qβ [114]. In *A. thaliana*, RPL5 and transcription factor IIIA bind Potato spindle tuber viroid (PSTVd) RNA to participate in the RNA synthesis of PSTVd [115]. Furthermore, a few RPs can bind viral noncoding RNA to maintain their viral functions. Epstein–Barr virus (EBV)-encoded RNA 1 (EBER-1), a noncoding RNA, recruiting human RPL22 mediates relocalization of RPL22 in EBV-infected cells, which is critical for EBV-associated tumorigenesis [116,117,118].

## 3. Antiviral Function of Ribosomal Proteins

Compared with RPs positive regulating viral infection, the reports on antiviral function of RPs are rare and appear recently in terms of time. Initially, it is known that the overall inhibition of host protein synthesis induced by poliovirus infection can be resisted by translation of RP mRNA and phosphorylation of RPS6 [119]. According to existing reports, there are two main antiviral mechanisms of RPs. First, RPs interact with viral proteins to inhibit directly virus transcription or translation. RPL9 binds phosphoprotein P (an essential cofactor of viral RNA polymerase) of Rabies virus (RABV) and translocates from nucleus to cytoplasm, inhibiting the initial stage of RABV transcription [120]. RPS10, 18S rRNA and lesser tRNAs bind to Nef protein of HIV-1 to from a complex, decreasing viral protein synthesis [121]. Second, RPs as immune factors activate antiviral defense signaling pathways. RPS20 inhibits CSFV replication in cells by modulating Toll-like receptor 3 (TLR3), which can activate immune responses [122]. In response to infection by Respiratory syncytial virus, RRL13a is released from 60S subunit and assemble an interferon-γ independent antiviral complex to suppress the translation of a specific viral mRNA (matrix protein M), which represents a novel paradigm in antiviral innate immunity [123]. In geminivirus nuclear shuttle protein (NSP)-interacting kinase (NIK)-mediated antiviral defense pathway in plants, RRL10 acts as an immediate downstream effector of the antiviral signaling, in which RRL10—a specific partner and substrate of NIK—is phosphorylated and redirects to the nucleus to modulate viral infection [124,125]. It is a new defense strategy of plant cells against viruses.

## 4. Applications and Prospective

Given wide-ranging functions of RPs on viral infection, RPs have huge therapeutic potential as targets. The ribosome inactivating proteins (RIPs) are RNA N-glycosidases that inhibit protein synthesis by selectively modifying large rRNA molecules and deactivating ribosomes [126], so some RIPs exhibit antiviral activity. RIP from *Phytolacca americana* is a famous representative, also known as Pokeweed antiviral protein (PAP) [127]. PAP serves as effective inhibitor of many animal and plant viruses, for instance, HIV-1, poliovirus, HSV-1, TMV and Tobacco etch virus, etc. [128,129,130]. The antiviral activity of PAP has been explored by many researchers, via conjugating a variety of monoclonal antibodies for antiviral therapeutics [131,132] and generating transgenic plants resistant to plant viruses [133]. The antiviral mechanism of PAP is poorly understood. It is proposed that PAP may inhibit virus translation by binding and depurinating capped viral RNAs [134]. However, PAP does not depurinate uncapped viral RNAs (such as TBSV) and IRES-containing RNA (such as poliovirus), but PAP inhibit translation of these viral RNAs [135]. The mode of ribosomal inactivation by PAP remains to be elucidated. Due to nonspecific inactivating to ribosomes, many RIPs, such as that from *Ricinus communis* and *Abrus precatorius*, are high toxicity to eukaryotic cells [136], which limits broad-spectrum application of RIPs in antiviral therapeutics.

Many RPs exhibit upregulating critical roles in the life cycle of various viruses, which makes them potential targets for design small molecule antiviral drugs, especially those RPs with reductions that are non-essential for ribosomal function to maintain cell cycle, for example, RPS25, RPS27, RPLP1, RPLP2, RPL3 and RPL18, deserve special attention. In these non-essential RPs, the range of viruses regulated by RPS27 is broad. RPS27 can regulate infection of fatal IVA, DENV, HCV and Sindbis virus [111], which increases its value as a target. Currently, the development of antiviral therapy with these RPs as targets is insufficient. The antivirals picolinic acid (PA) and fusaric acid (FU) are the successful examples. PA and FU exhibit effective antiviral and preventive activities for a series of DNA and RNA virus by disrupting ZFP domain of RPS27 and inactivating this RP [111]. Furthermore, many viruses encode ZFP-containing viral proteins for replication, such as HIV-1 Np7, HSV-1 ICPO, IVA M1, HCV NS2 and Reoviruses ρ-3, etc., while PA and FU also disrupt essential viral ZFPs and render pathogenic virus inactive [111], which enhances the antiviral effect of PA and FU. RPS25 controls viral IRES and ribosome shunt-mediated translation [31], and RPL18 also has a broader range of viruses to regulate (Table 1), which endows the two RPs great potential for antiviral application. Unfortunately, there is no correlated report. RNAi and CRISPR/Cas9-based strategies are promising approaches to target RP genes. For potential target RPs, although their depletions are non-essential for basic ribosomal function in the experiments, it is suggested knockdown and suppression of these RPs rather than knocked out or mutation in application. Due to the multiformity of RPs in cells [4,5,6,7], absence and mutation of any RP might lead to exceptional responses of cells to physiological and non-viral pathological stimuli.

Moreover, many RPs exhibit dramatically higher levels of expression in cells where virus propagate rapidly, such as RPL4 in EBV-infected cells [94] and RPS27a in Hepatitis B virus-infected cells [137]. These RPs with high expression can be considered as targets of antiviral drug carriers for specific tissue/cell drug delivery. For example, we envisage a project in which the anti-EBV drug is encapsulated by the diblock copolymer to form nanoparticles (NPs), and then the surface of NPs is conjugated by RPL4 monoclonal antibody (mAb) or Fab-fragment of RPL4 mAb. The produced polymeric NPs drug will be responsive to the cells with more RPL4 to trigger drug targeting delivery, resulting in more effective clinical antiviral therapy.

RPs with antiviral function can also be directly utilized. HP (2-20), an antibacterial sequence with a membrane-disruptive property from RPL1 of *Helicobacter pylori*, is developed the novel virus–cell fusion inhibitory peptide to defense virus infection after several amino acid sites substitution [138]. Interest in RPs involved in viral infection by scientists and physicians have markedly increased, but we are still in an early stage of targeting RPs for therapeutic applications.

## 5. Conclusions

This review provides an overview about the positive and negative roles of RPs on viral infection control in cells. More and more information is available on the critical roles and precise modes of RPs in virus infections, thus, RPs are viewed as being of immense importance for preventing deathly viral diseases. As our understanding of biological function of RPs increases, antiviral design principles and strategies targeting RPs will also keep pace, and more medicaments, like PA and FU, will appear. In general, RPs provide a new platform for antiviral therapy development, but at present, antiviral therapeutics based on RPs is limited, and many challenges remain to be overcome. Exploring novel antiviral medicament and strategy targeting RPs will be the guides for further study in this field.

## Figures and Tables

**Table 1:** The characterized roles of the interactions between RPs and viral proteins on positive regulating viral infection.

No.	RP	Virus	Interacting Viral Proteins	Structural (s) or Nonstructural (ns) Protein	Function of Interaction	Ref.
1.	RPS19	Sin nombre hantavirus (SNV)	N	S	Facilitating N-mediated translation initiation mechanism	[76,77,78]
2.	RPL18	Cauliflower mosaic virus (CaMV)	P6	NS	Translational transactivation	[79,80]
		Rice stripe tenuivirus (RSV)	N	S	Promoting viral translation and replication	[82]
		Dengue virus (DENV)	NS1	NS	Required for viral translation and replication	[83]
		Potato virus A (PVA)	HCPro	NS	Forming complexes to relieve viral RNA translational repression from RISC	[88]
		Infectious bursal disease virus (IBDV)	VP3	S	Enhancing viral replication	[92]
3.	RPS2 (RPSA)	DENV	Envelope protein E	S	Viral membrane receptor	[86]
		Yellow fever virus (YFV)	Envelope protein E	S	Viral membrane receptor	[86]
		Tobacco etch virus (TEV)	HCPro	NS	Viral receptor in the transmission process	[91]
4.	RPL4	IBDV	VP3	S	Modulation of IBDV replication	[93]
		Epstein-Barr virus (EBV)	EBNA1	NS	Establishing persistent B-lymphoblastoid cell infection	[94]
5.	RPL7	Human immunodeficiency virus type 1 (HIV-1)	Gag	S	As a helper contributing to the start of Gag assembly	[84]
		White spot syndrome virus (WSSV)	VP51	S	Involving in WSSV infection	[95]
6.	RPS6	Kaposi’s sarcoma-associated herpesvirus (KSHV)	LANA	NS	Maintaining LANA stability	[71]
7.	RPS11	Cucumber mosaic virus (CMV)	2b	NS	Affecting viral replication, infection and gene silencing suppressor activity	[96]
8.	RPS27a	EBV	LMP1	NS	Stabilizing of LMP1 to enhance viral proliferation and invasion	[97]
9.	RPL6	Human T-cell leukemia virus type 1 (HTLV-1)	Tax	NS	Facilitating production of viral particles	[98]
10.	RPL9	Mouse mammary tumor virus (MMTV)	Gag	S	Efficient virus particle assembly	[99]
11.	RPL10 (P0)	PVA	VPg	NS	Enhancing viral translation	[90]
12.	RPL13	CaMV	P6	NS	Reinitiation of viral translation	[80]
13.	RPL22	Herpes simplex virus 1 (HSV-1)	ICP4	NS	Regulatory of virus DNA synthesis and late gene expression in nucleus	[85]
14.	RPL24	TEV	P1	NS	Stimulating translation of viral proteins	[75]

## References

[1] Wool I.G. (1979). The structure and function of eukaryotic ribosomes. Annu. Rev. Biochem..

[2] Wilson D.N., Doudna Cate J.H. (2012). The structure and function of the eukaryotic ribosome. Cold Spring Harb. Perspect. Biol..

[3] Fromont-Racine M., Senger B., Saveanu C., Fasiolo F. (2003). Ribosome assembly in eukaryotes. Gene.

[4] Xu X., Xiong X., Sun Y. (2016). The role of ribosomal proteins in the regulation of cell proliferation, tumorigenesis, and genomic integrity. Sci. China Life Sci..

[5] Chen F.W., Ioannou Y.A. (1999). Ribosomal proteins in cell proliferation and apoptosis. Int. Rev. Immunol..

[6] Bhavsar R.B., Makley L.N., Tsonis P.A. (2010). The other lives of ribosomal proteins. Hum. Genom..

[7] De Las Heras-Rubio A., Perucho L., Paciucci R., Vilardell J., Leonart M.E. (2014). Ribosomal proteins as novel players in tumorigenesis. Cancer Metastasis Rev..

[8] Lee A.S., Burdeinick-Kerr R., Whelan S.P. (2013). A ribosome-specialized translation initiation pathway is required for cap-dependent translation of vesicular stomatitis virus mRNAs. Proc. Natl. Acad. Sci. USA.

[9] Simonin D., Diaz J.J., Massé T., Madjar J.J. (1997). Persistence of ribosomal protein synthesis after infection of HeLa cells by herpes simplex virus type 1. J. Gen. Virol..

[10] Greco A., Laurent A.M., Madjar J.J. (1997). Repression of beta-actin synthesis and persistence of ribosomal protein synthesis after infection of HeLa cells by herpes simplex virus type 1 infection are under translational control. Mol. Gen. Genet..

[11] Jackson R.J., Hellen C.U., Pestova T.V. (2010). The mechanism of eukaryotic translation initiation and principles of its regulation. Nat. Rev. Mol. Cell Biol..

[12] Plotch S.J., Bouloy M., Ulmanen I., Krug R.M. (1981). A unique cap(m7GpppXm)-dependent influenza virion endonuclease cleaves capped RNAs to generate the primers that initiate viral RNA transcription. Cell.

[13] Ramirez B.C., Garcin D., Calvert L.A., Kolakofsky D., Haenni A. (1995). Capped nonviral sequences at the 5’ end of the mRNAs of rice hoja blanca virus RNA4. J. Virol..

[14] Pelletier J., Sonenberg N. (1988). Internal initiation of translation of eukaryotic mRNA directed by a sequence derived from poliovirus RNA. Nature.

[15] Jang S.K., Kräusslich H.G., Nicklin M.J., Duke G.M., Palmenberg A.C., Wimmer E. (1988). A segment of the 5’ nontranslated region of encephalomyocarditis virus RNA directs internal entry of ribosomes during in vitro translation. J. Virol..

[16] Otto G.A., Puglisi J.D. (2004). The pathway of HCV IRES-mediated translation initiation. Cell.

[17] Bushell M., Sarnow P. (2002). Hijacking the translation apparatus by RNA viruses. J. Cell Biol..

[18] Muhs M., Yamamoto H., Ismer J., Takaku H., Nashimoto M., Uchiumi T., Nakashima N., Mielke T., Hildebrand P.W., Nierhaus K.H. (2011). Structural basis for the binding of IRES RNAs to the head of the ribosomal 40S subunit. Nucleic Acids Res..

[19] Kieft J.S. (2008). Viral IRES RNA structures and ribosome interactions. Trends Biochem. Sci..

[20] Pestova T.V., Lomakin I.B., Hellen C.U. (2004). Position of the CrPV IRES on the 40S subunit and factor dependence of IRES/80S ribosome assembly. EMBO Rep..

[21] Deniz N., Lenarcic E.M., Landry D.M., Thompson S.R. (2009). Translation initiation factors are not required for Dicistroviridae IRES function in vivo. RNA.

[22] Pestova T.V., Shatsky I.N., Fletcher S.P., Jackson R.J., Hellen C.U. (1998). A prokaryotic-like mode of cytoplasmic eukaryotic ribosome binding to the initiation codon during internal translation initiation of hepatitis C and classical swine fever virus RNAs. Genes Dev..

[23] Fraser C.S., Doudna J.A. (2007). Structural and mechanistic insights into hepatitis C viral translation initiation. Nat. Rev. Microbiol..

[24] Fitzgerald K.D., Semler B.L. (2009). Bridging IRES elements in mRNAs to the eukaryotic translation apparatus. Biochim. Biophys. Acta.

[25] Petrov A., Grosely R., Chen J., O’Leary S.E., Puglisi J.D. (2016). Multiple parallel pathways of translation initiation on the CrPV IRES. Mol. Cell..

[26] Spahn C.M., Jan E., Mulder A., Grassucci R.A., Sarnow P., Frank J. (2004). Cryo-EM visualization of a viral internal ribosome entry site bound to human ribosomes: The IRES functions as an RNA-based translation factor. Cell.

[27] Schuler M., Connell S.R., Lescoute A., Giesebrecht J., Dabrowski M., Schroeer B., Mielke T., Penczek P.A., Westhof E., Spahn C.M. (2006). Structure of the ribosome-bound cricket paralysis virus IRES RNA. Nat. Struct. Mol. Biol..

[28] Spahn C.M., Kieft J.S., Grassucci R.A., Penczek P.A., Zhou K., Doudna J.A., Frank J. (2001). Hepatitis C virus IRES RNA-induced changes in the conformation of the 40s ribosomal subunit. Science.

[29] Fukushi S., Okada M., Stahl J., Kageyama T., Hoshino F.B., Katayama K. (2001). Ribosomal protein S5 interacts with the internal ribosomal entry site of hepatitis C virus. J. Biol Chem..

[30] Bhat P., Shwetha S., Sharma D.K., Joseph A.P., Srinivasan N., Das S. (2015). The beta hairpin structure within ribosomal protein S5 mediates interplay between domains II and IV and regulates HCV IRES function. Nucleic Acids Res..

[31] Hertz M.I., Landry D.M., Willis A.E., Luo G., Thompson S.R. (2013). Ribosomal protein S25 dependency reveals a common mechanism for diverse internal ribosome entry sites and ribosome shunting. Mol. Cell Biol..

[32] Armache J.P., Jarasch A., Anger A.M., Villa E., Becker T., Bhushan S., Jossinet F., Habeck M., Dindar G., Franckenberg S. (2010). Localization of eukaryote-specific ribosomal proteins in a 5.5-A cryo-EM map of the 80S eukaryotic ribosome. Proc. Natl. Acad. Sci. USA.

[33] Rabl J., Leibundgut M., Ataide S.F., Haag A., Ban N. (2011). Crystal structure of the eukaryotic 40S ribosomal subunit in complex with initiation factor 1. Science.

[34] Nishiyama T., Yamamoto H., Uchiumi T., Nakashima N. (2007). Eukaryotic ribosomal protein RPS25 interacts with the conserved loop region in a dicistroviral intergenic internal ribosome entry site. Nucleic Acids Res..

[35] Galkin O., Bentley A.A., Gupta S., Compton B.A., Mazumder B., Kinzy T.G., Merrick W.C., Hatzoglou M., Pestova T.V., Hellen C.U. (2007). Roles of the negatively charged N-terminal extension of Saccharomyces cerevisiae ribosomal protein S5 revealed by characterization of a yeast strain containing human ribosomal protein S5. RNA.

[36] Landry D.M., Hertz M.I., Thompson S.R. (2009). RPS25 is essential for translation initiation by the Dicistroviridae and hepatitis C viral IRESs. Genes Dev..

[37] Carvajal F., Vallejos M., Walters B., Contreras N., Hertz M.I., Olivares E., Cáceres C.J., Pino K., Letelier A., Thompson S.R. (2016). Structural domains within the HIV-1 mRNA and the ribosomal protein S25 influence cap-independent translation initiation. FEBS J..

[38] Majzoub K., Hafirassou M.L., Meignin C., Goto A., Marzi S., Fedorova A., Verdier Y., Vinh J., Hoffmann J.A., Martin F. (2014). RACK1 controls IRES-mediated translation of viruses. Cell.

[39] Huang J.Y., Su W.C., Jeng K.S., Chang T.H., Lai M.M. (2012). Attenuation of 40S ribosomal subunit abundance differentially affects host and HCV translation and suppresses HCV replication. PLoS Pathog..

[40] Laletina E., Graifer D., Malygin A., Ivanov A., Shatsky I., Karpova G. (2006). Proteins surrounding hairpin IIIe of the hepatitis C virus Internal Ribosome Entry Site on the human 40S ribosomal subunit. Nucleic Acids Res..

[41] Babaylova E., Graifer D., Malygin A., Stahl J., Shatsky I., Karpova G. (2009). Positioning of subdomain IIId and apical loop of domain II of the hepatitis C IRES on the human 40S ribosome. Nucleic Acids Res..

[42] Futterer J., Kiss-Laszlo Z., Hohn T. (1993). Nonlinear ribosome migration on cauliflower mosaic virus 35S RNA. Cell.

[43] Pooggin M.M., Ryabova L.A., He X., Futterer J., Hohn T. (2006). Mechanism of ribosome shunting in Rice tungro bacilliform pararetrovirus. RNA.

[44] Curran J., Kolakofsky D. (1988). Scanning independent ribosomal initiation of the Sendai virus X protein. EMBO J..

[45] Yueh A., Schneider R.J. (1996). Selective translation initiation by ribosome jumping in adenovirus-infected and heat-shocked cells. Genes Dev..

[46] Yueh A., Schneider R.J. (2000). Translation by ribosome shunting on adenovirus and hsp70 mRNAs facilitated by complementarity to 18S rRNA. Genes Dev..

[47] Sherrill K.W., Lloyd R.E. (2008). Translation of cIAP2 mRNA is mediated exclusively by a stress-modulated ribosome shunt. Mol. Cell. Biol..

[48] Atkins J.F., Loughran G., Bhatt P.R., Firth A.E., Baranov P.V. (2016). Ribosomal frameshifting and transcriptional slippage: From genetic steganography and cryptography to adventitious use. Nucleic Acids Res..

[49] Jacks T., Power M.D., Masiarz F.R., Luciw P.A., Barr P.J., Varmus H.E. (1988). Characterization of ribosomal frameshifting in HIV-1 gag-pol expression. Nature.

[50] Wilson W., Braddock M., Adams S.E., Rathjen P.D., Kingsman S.M., Kingsman A.J. (1988). HIV expression strategies: Ribosomal frameshifting is directed by a short sequence in both mammalian and yeast systems. Cell.

[51] Shehu-Xhilaga M., Crowe S.M., Mak J. (2001). Maintenance of the Gag/Gag-Pol ratio is important for human immunodeficiency virus type 1 RNA dimerization and viral infectivity. J. Virol..

[52] Dulude D., Berchiche Y.A., Gendron K., Brakier-Gingras L., Heveker N. (2006). Decreasing the frameshift efficiency translates into an equivalent reduction of the replication of the human immunodeficiency virus type 1. Virology.

[53] Wang X., Xuan Y., Han Y., Ding X., Ye K., Yang F., Gao P., Goff S.P., Gao G. (2019). Regulation of HIV-1 Gag-Pol expression by shiftless, an inhibitor of programmed -1 ribosomal frameshifting. Cell.

[54] Peltz S.W., Hammell A.B., Cui Y., Yasenchak J., Puljanowski L., Dinman J.D. (1999). Ribosomal protein L3 mutants alter translational fidelity and promote rapid loss of the yeast killer virus. Mol. Cell. Biol..

[55] Kaerlein M., Horak I. (1978). Identification and characterization of ribosomal proteins phosphorylated in vaccinia-virus-infected HeLa cells. Eur. J. Biochem..

[56] Beaud G., Masse T., Madjar J.J., Leader D.P. (1989). Identification of induced protein kinase activities specific for the ribosomal proteins uniquely phosphorylated during infection of HeLa cells with vaccinia virus. FEBS Lett..

[57] Meyuhas O. (2008). Physiological roles of ribosomal protein S6: One of its kind. Int. Rev. Cell Mol. Biol..

[58] Kaerlein M., Horak I. (1976). Phosphorylation of ribosomal proteins in HeLa cells infected with vaccinia virus. Nature.

[59] Kennedy I.M., Stevely W.S., Leader D.P. (1981). Phosphorylation of ribosomal proteins in hamster fibroblasts infected with pseudorabies virus or herpes simplex virus. J. Virol..

[60] Kennedy I.M., Leader D.P. (1981). Increased phosphorylation of ribosomal protein S6 in hamster fibroblasts transformed by polyoma virus and simian virus 40. Biochem. J..

[61] Decker S. (1981). Phosphorylation of ribosomal protein S6 in avian sarcoma virus-transformed chicken embryo fibroblasts. Proc. Natl. Acad. Sci. USA.

[62] Blenis J., Erikson R.L. (1984). Phosphorylation of the ribosomal protein S6 is elevated in cells transformed by a variety of tumor viruses. J. Virol..

[63] Katan M., McGarvey M.J., Stevely W.S., Leader D.P. (1986). The phosphorylation of ribosomal protein S6 by protein kinases from cells infected with pseudorabies virus. Biochem. J..

[64] Jakubowicz T., Leader D.P. (1987). Activation of a ribosomal protein S6 kinase in mouse fibroblasts during infection with herpesvirus. Eur. J. Biochem..

[65] Banham A.H., Leader D.P., Smith G.L. (1993). Phosphorylation of ribosomal proteins by the vaccinia virus B1R proteinkinase. FEBS Lett..

[66] Beaud G., Sharif A., Topa-Massé A., Leader D.P. (1994). Ribosomal protein S2/Sa kinase purified from HeLa cells infected with vaccinia virus corresponds to the B1R protein kinase and phosphorylates in vitro the viral ssDNA-binding protein. J. Gen. Virol..

[67] Maller J.L., Foulkes J.G., Erikson E., Baltimore D. (1985). Phosphorylation of ribosomal protein S6 on serine after microinjection of the Abelson murine leukemia virus tyrosine-specific protein kinase into *Xenopus oocytes*. Proc. Natl. Acad. Sci. USA.

[68] Meyuhas O. (2015). Ribosomal protein S6 phosphorylation: Four decades of research. Int. Rev. Cell Mol. Biol..

[69] Kennedy I.M., Leader D.P., Stevely W.S. (1984). The phosphorylation of ribosomal protein S6 in hamster fibroblasts infected with pseudorabies virus inactivated by ultraviolet radiation. J. Gen. Virol..

[70] Montgomery S.A., Berglund P., Beard C.W., Johnston R.E. (2006). Ribosomal protein S6 associates with alphavirus nonstructural protein 2 and mediates expression from alphavirus messages. J. Virol..

[71] Chen W., Dittmer D.P. (2011). Ribosomal protein S6 interacts with the latency-associated nuclear antigen of Kaposi’s sarcoma-associated herpesvirus. J. Virol..

[72] Rajamäki M.L., Xi D., Sikorskaite-Gudziuniene S., Valkonen J., Whitham S.A. (2017). Differential requirement of the ribosomal protein S6 and ribosomal protein S6 kinase for plant-virus accumulation and interaction of S6 kinase with potyviral VPg. Mol. Plant. Microbe Interact..

[73] Yang C., Zhang C., Dittman J.D., Whitham S.A. (2009). Differential requirement of ribosomal protein S6 by plant RNA viruses with different translation initiation strategies. Virology.

[74] Jefferson M., Donaszi-Ivanov A., Pollen S., Dalmay T., Saalbach G., Powell P.P. (2014). Host factors that interact with the pestivirus N-terminal protease, N^pro^, are components of the ribonucleoprotein complex. J. Virol..

[75] Martínez F., Daròs J.A. (2014). Tobacco etch virus protein P1 traffics to the nucleolus and associates with the host 60S ribosomal subunits during infection. J. Virol..

[76] Ganaie S.S., Haque A., Cheng E., Bonny T.S., Salim N.N., Mir M.A. (2014). Ribosomal protein S19-binding domain provides insights into hantavirus nucleocapsid protein-mediated translation initiation mechanism. Biochem. J..

[77] Cheng E., Haque A., Rimmer M.A., Hussein I.T., Sheema S., Little A., Mir M.A. (2011). Characterization of the Interaction between hantavirus nucleocapsid protein (N) and ribosomal protein S19 (RPS19). J. Biol. Chem..

[78] Haque A., Mir M.A. (2010). Interaction of hantavirus nucleocapsid protein with ribosomal protein S19. J. Virol..

[79] Leh V., Yot P., Keller M. (2000). The cauliflower mosaic virus translational transactivator interacts with the 60S ribosomal subunit protein L18 of *Arabidopsis thaliana*. Virology.

[80] Bureau M., Leh V., Haas M., Geldreich A., Ryabova L., Yot P., Keller M. (2004). P6 protein of Cauliflower mosaic virus, a translation reinitiator, interacts with ribosomal protein L13 from *Arabidopsis thaliana*. J. Gen. Virol..

[81] Spurgers K.B., Alefantis T., Peyser B.D., Ruthel G.T., Bergeron A.A., Costantino J.A., Enterlein S., Kota K.P., Boltz R.C., Aman M.J. (2010). Identification of essential filovirion-associated host factors by serial proteomic analysis and RNAi screen. Mol. Cell. Proteomics.

[82] Li S., Li X., Zhou Y. (2018). Ribosomal protein L18 is an essential factor that promote rice stripe virus accumulation in small brown planthopper. Virus Res..

[83] Cervantes-Salazar M., Angel-Ambrocio A.H., Soto-Acosta R., Bautista-Carbajal P., Hurtado-Monzon A.M., Alcaraz-Estrada S.L., Ludert J.E., Del Angel R.M. (2015). Dengue virus NS1 protein interacts with the ribosomal protein RPL18: This interaction is required for viral translation and replication in Huh-7 cells. Virology.

[84] Mekdad H.E., Boutant E., Karnib H., Biedma M.E., Sharma K.K., Malytska I., Laumond G., Roy M., Réal E., Paillart J.C. (2016). Characterization of the interaction between the HIV-1 Gag structural polyprotein and the cellular ribosomal protein L7 and its implication in viral nucleic acid remodeling. Retrovirology.

[85] Leopardi R., Roizman B. (1996). Functional interaction and colocalization of the herpes simplex virus 1 major regulatory protein ICP4 with EAP, a nucleolar-ribosomal protein. Proc. Natl. Acad. Sci. USA.

[86] Zidane N., Ould-Abeih M.B., Petit-Topin I., Bedouelle H. (2012). The folded and disordered domains of human ribosomal protein SA have both idiosyncratic and shared functions as membrane receptors. Biosci. Rep..

[87] Plisson C., Drucker M., Blanc S., German-Retana S., Le Gall O., Thomas D., Bron P. (2003). Structural characterization of HC-Pro, a plant virus multifunctional protein. J. Biol. Chem..

[88] Ivanov K.I., Eskelin K., Bašić M., De S., Lõhmus A., Varjosalo M., Mäkinen K. (2016). Molecular insights into the function of the viral RNA silencing suppressor HCPro. Plant J..

[89] Ala-Poikela M., Goytia E., Haikonen T., Rajamäki M.L., Valkonen J.P. (2011). Helper component proteinase of the genus *Potyvirus* is an interaction partner of translation initiation factors eIF(iso)4E and eIF4E and contains a 4E binding motif. J. Virol..

[90] Hafrén A., Eskelin K., Mäkinen K. (2013). Ribosomal protein P0 promotes Potato virus A infection and functions in viral translation together with VPg and eIF(iso)4E. J. Virol..

[91] Fernández-Calvino L., Goytia E., López-Abella D., Giner A., Urizarna M., Vilaplana L., López-Moya J.J. (2010). The helper-component protease transmission factor of tobacco etch potyvirus binds specifically to an aphid ribosomal protein homologous to the laminin receptor precursor. J. Gen. Virol..

[92] Wang B., Duan X., Fu M., Liu Y., Wang Y., Li X., Cao H., Zheng S.J. (2018). The association of ribosomal protein L18 (RPL18) with infectious bursal disease virus viral protein VP3 enhances viral replication. Virus Res..

[93] Chen Y., Lu Z., Zhang L., Gao L., Wang N., Gao X., Wang Y., Li K., Gao Y., Cui H. (2016). Ribosomal protein L4 interacts with viral protein VP3 and regulates the replication of infectious bursal disease virus. Virus Res..

[94] Shen C.L., Liu C.D., You R.I., Ching Y.H., Liang J., Ke L., Chen Y.L., Chen H.C., Hsu H.J., Liou J.W. (2016). Ribosome protein L4 is essential for Epstein-Barr virus nuclear antigen 1 function. Proc. Natl. Acad. Sci. USA.

[95] Liu Q., Ma F., Guan G., Wang X., Li C., Huang J. (2015). White spot syndrome virus VP51 interact with ribosomal protein L7 of *Litopenaeusvannamei*. Fish. Shellfish Immunol..

[96] Wang R., Du Z., Bai Z., Liang Z. (2017). The interaction between endogenous 30S ribosomal subunit protein S11 and Cucumber mosaic virus LS2b protein affects viral replication, infection and gene silencing suppressor activity. PLoS ONE.

[97] Hong S.W., Kim S.M., Jin D.H., Kim Y.S., Hur D.Y. (2017). RPS27a enhances EBV-encoded LMP1-mediated proliferation and invasion by stabilizing of LMP1. Biochem. Biophys. Res. Commun..

[98] Wang J., Yang X., Zhou P., Han H. (2002). Cloning of mouse genomic ribosomal protein L6 gene and analysis of its promoter. Biochim. Biophys. Acta.

[99] Beyer A.R., Bann D.V., Rice B., Pultz I.S., Kane M., Goff S.P., Golovkina T.V., Parent L.J. (2013). Nucleolar trafficking of the mouse mammary tumor virus gag protein induced by interaction with ribosomal protein L9. J. Virol..

[100] Campos R.K., Wong B., Xie X., Lu Y.F., Shi P.Y., Pompon J., Garcia-Blanco M.A., Bradrick S.S. (2017). RPLP1 and RPLP2 are essential flavivirus host factors that promote early viral protein accumulation. J. Virol..

[101] Kenney S.P., Meng X.J. (2015). The lysine residues within the human ribosomal protein S17 sequence naturally inserted into the viral nonstructural protein of a unique strain of hepatitis E virus are important for enhanced virus replication. J. Virol..

[102] Schatz O., Oft M., Dascher C., Schebesta M., Rosorius O., Jaksche H., Dobrovnik M., Bevec D., Hauber J. (1998). Interaction of the HIV-1 rev cofactor eukaryotic initiation factor 5A with ribosomal protein L5. Proc. Natl. Acad. Sci. USA.

[103] Lam Y.W., Trinkle-Mulcahy L., Lamond A.I. (2005). The nucleolus. J. Cell Sci..

[104] Grummt I. (2003). Life on a planet of its own: Regulation of RNA polymerase I transcription in the nucleolus. Genes Dev..

[105] Cawood R., Harrison S.M., Dove B.K., Reed M.L., Hiscox J.A. (2007). Cell cycle dependent nucleolar localization of the coronavirus nucleocapsid protein. Cell Cycle.

[106] Chen H., Wurm T., Britton P., Brooks G., Hiscox J.A. (2002). Interaction of the coronavirus nucleoprotein with nucleolar antigens and the host cell. J. Virol..

[107] Dove B.K., You J.H., Reed M.L., Emmett S.R., Brooks G., Hiscox J.A. (2006). Changes in nucleolar morphology and proteins during infection with the coronavirus infectious bronchitis virus. Cell. Microbiol..

[108] Emmott E., Smith C., Emmett S.R., Dove B.K., Hiscox J.A. (2010). Elucidation of the avian nucleolar proteome by quantitative proteomics using SILAC and changes in cells infected with the coronavirus infectious bronchitis virus. Proteomics.

[109] Fernandez-Pol J.A., Hamilton P.D., Klos D.J. (2001). Essential viral and cellular zinc and iron containing metalloproteins as targets for novel antiviral and anticancer agents: Implications for prevention and therapy of viral diseases and cancer. Anticancer Res..

[110] Fernandez-Pol J.A., Klos D.J., Hamilton P.D. (2005). Genomics, proteomics and cancer: Specific ribosomal, mitochondrial, and tumor reactive proteins can be used as biomarkers for early detection of breast cancer in serum. Cancer Genom. Proteom..

[111] Fernandez-Pol J.A. (2011). Conservation of multifunctional ribosomal protein metallopanstimulin-1 (RPS27) through complex evolution demonstrates its key role in growth regulation in Archaea, eukaryotic cells, DNA repair, translation and viral replication. Cancer Genom. Proteom..

[112] Karlas A., Machuy N., Shin Y., Pleissner K.P., Artarini A., Heuer D., Becker D., Khalil H., Ogilvie L.A., Hess S. (2010). Genome-wide RNAi screen identifies human host factors crucial for influenza virus replication. Nature.

[113] Cherry S., Doukas T., Armknecht S., Whelan S., Wang H., Sarnow P., Perrimon N. (2005). Genome-wide RNAi screen reveals a specific sensitivity of IRES-containing RNA viruses to host translation inhibition. Genes Dev..

[114] Takeshita D., Yamashita S., Tomita K. (2014). Molecular insights into replication initiation by Qβ replicase using ribosomal protein S1. Nucleic Acids Res..

[115] Eiras M., Nohales M.A., Kitajima E.W., Flores R., Daròs J.A. (2011). Ribosomal protein L5 and transcription factor IIIA from *Arabidopsis thaliana* bind in vitro specifically Potato spindle tuber viroid RNA. Arch. Virol..

[116] Houmani J.L., Davis C.I., Ruf I.K. (2009). Growth-promoting properties of Epstein-Barr virus EBER-1 RNA correlate with ribosomal protein L22 binding. J. Virol..

[117] Fok V., Mitton-Fry R.M., Grech A., Steitz J.A. (2006). Multiple domains of EBER 1, an Epstein-Barr virus noncoding RNA, recruit human ribosomal protein L22. RNA.

[118] Houmani J.L., Ruf I.K. (2009). Clusters of basic amino acids contribute to RNA binding and nucleolar localization of ribosomal protein L22. PLoS ONE.

[119] Cardinali B., Fiore L., Campioni N., De Dominicis A., Pierandrei-Amaldi P. (1999). Resistance of ribosomal protein mRNA translation to protein synthesis shutoff induced by poliovirus. J. Virol..

[120] Li Y., Dong W., Shi Y., Deng F., Chen X., Wan C., Zhou M., Zhao L., Fu Z., Peng G. (2016). Rabies virus phosphoprotein interacts with ribosomal protein L9 and affects rabies virus replication. Virology.

[121] Abbas W., Dichamp I., Herbein G. (2012). The HIV-1 Nef protein interacts with two components of the 40S small ribosomal subunit, the RPS10 protein and the 18S rRNA. Virol. J..

[122] Lv H., Dong W., Qian G., Wang J., Li X., Cao Z., Lv Q., Wang C., Guo K., Zhang Y. (2017). uS10, a novel Npro-interacting protein, inhibits classical swine fever virus replication. J. Gen. Virol..

[123] Mazumder B., Poddar D., Basu A., Kour R., Verbovetskaya V., Barik S. (2014). Extraribosomal l13a is a specific innate immune factor for antiviral defense. J. Virol..

[124] Carvalho C.M., Santos A.A., Pires S.R., Rocha C.S., Saraiva D.I., Machado J.P., Mattos E.C., Fietto L.G., Fontes E.P. (2008). Regulated nuclear trafficking of rpL10A mediated by NIK1 represents a defense strategy of plant cells against virus. PLoS Pathog..

[125] Rocha C.S., Santos A.A., Machado J.P., Fontes E.P. (2008). The ribosomal protein L10/QM-like protein is a component of the NIK-mediated antiviral signaling. Virology.

[126] Peumans W.J., Hao Q., van Damme E.J. (2001). Ribosome-inactivating proteins from plants: More than RNA N-glycosidases?. FASEB J..

[127] Irvin J.D. (1975). Purification and partial characterization of the antiviral protein from *Phytolaccaamericana* which inhibits eukaryotic protein synthesis. Arch. Biochem. Biophys..

[128] Domashevskiy A.V., Goss D.J. (2015). Pokeweed antiviral protein, a ribosome inactivating protein: Activity, inhibition and prospects. Toxins.

[129] Rajamohan F., Venkatachalam T.K., Irvin J.D., Uckun F.M. (1999). Pokeweed antiviral protein isoforms PAP-I, PAP-II, and PAP-III depurinate RNA of human immunodeficiency virus (HIV)-1. Biochem. Biophys. Res. Commun..

[130] Uckun F.M., Rajamohan F., Pendergrass S., Ozer Z., Waurzyniak B., Mao C. (2003). Structure-based design and engineering of a nontoxic recombinant pokeweed antiviral protein with potent anti-human immunodeficiency virus activity. Antimicrob. Agents Chemother..

[131] Zarling J.M., Moran P.A., Haffar O., Sias J., Richman D.D., Spina C.A., Myers D.E., Kuebelbeck V., Ledbetter J.A., Uckun F.M. (1990). Inhibition of HIV replication by pokeweed antiviral protein targeted to CD4+ cells by monoclonal antibodies. Nature.

[132] Erice A., Balfour H.H., Myers D.E., Leske V.L., Sannerud K.J., Kuebelbeck V., Irvin J.D., Uckun F.M. (1993). Anti-human immunodeficiency virus type 1 activity of an anti-CD4 immunoconjugate containing pokeweed antiviral protein. Antimicrob. Agents Chemother..

[133] Lodge J.K., Kaniewski W.K., Tumer N.E. (1993). Broad-spectrum virus resistance in transgenic plants expressing pokeweed antiviral protein. Proc. Natl. Acad. Sci. USA.

[134] Hudak K.A., Bauman J.D., Tumer N.E. (2002). Pokeweed antiviral protein binds to the cap structure of eukaryotic mRNA and depurinates the mRNA downstream of the cap. RNA.

[135] Vivanco J.M., Tumer N.E. (2003). Translation inhibition of capped and uncapped viral RNAs mediated by ribosome-inactivating proteins. Phytopathology.

[136] Olsnes S. (2004). The history of ricin, abrin and related toxins. Toxicon.

[137] Fatima G., Mathan G., Kumar V. (2012). The HBx protein of hepatitis B virus regulates the expression, intracellular distribution and functions of ribosomal protein S27a. J. Gen. Virol..

[138] Woo E.R., Lee D.G., Chang Y.S., Park Y., Hahm K.S. (2002). Virus-cell fusion inhibitory activity of novel analogue peptides based on the HP (2–20) derived from N-terminus of *Helicobacter pylori* ribosomal protein L1. Protein Pept. Lett..

